# Rare Case of Community-Acquired Endocarditis Caused by *Neisseria meningitidis* Assessed by Clinical Metagenomics

**DOI:** 10.3389/fcvm.2019.00112

**Published:** 2019-08-06

**Authors:** Vassili Choutko, Vladimir Lazarevic, Nadia Gaïa, Myriam Girard, Gesuele Renzi, Stefano Leo, Peter M. Keller, Christoph Huber, Jacques Schrenzel

**Affiliations:** ^1^Service of General Internal Medicine, Geneva University Hospitals (HUG), Geneva, Switzerland; ^2^Genomic Research Laboratory, Service of Infectious Diseases, Geneva University Hospitals, Geneva University, Geneva, Switzerland; ^3^Bacteriology Laboratory, Service of Laboratory Medicine, Geneva University Hospitals, Geneva, Switzerland; ^4^Service of Laboratory Medicine, National Reference Center on Meningococci, Geneva University Hospitals, Geneva, Switzerland; ^5^Institute for Infectious Diseases, University of Bern, Bern, Switzerland; ^6^Department of Cardiovascular Surgery, Geneva University Hospitals, Geneva, Switzerland

**Keywords:** next-generation sequencing, cardiac valve, endocarditis, culture-negative infection, clinical metagenomics, *Neisseria meningitidis*

## Abstract

The most common causes of infective endocarditis (IE) are *Staphylococcus, Streptococcus, Enterococcus*, and HACEK-related organisms. In 15–30% of the IE cases, standard blood cultures remain sterile. We aimed at identifying the causative agent of a blood-culture-negative IE by whole metagenome shotgun sequencing (WMGS). A 54-year old woman diagnosed with community-onset pneumonia by a general practitioner, was admitted with dyspnea, cough and fever. The patient's blood cultures were repeatedly negative. The transesophageal echocardiography and transthoracic echocardiography showed an echo density on the left coronary leaflet of the aortic valve and signs suggestive of a ruptured abscess of the mitro-aortic junction. The patient underwent a semi-urgent aortic valve replacement by a mechanical prosthetic valve. We extracted DNA from the surgically-removed fresh valve tissue. The extraction procedure included bacterial/fungal DNA enrichment procedure. Nextera XT library prepared from the valve DNA extract was sequenced (2 × 250) on an Illumina MiSeq instrument. Sequence reads were mapped against bacterial genomic sequences, 16S rRNA genes and clade-specific taxonomic markers. Most of the 103,136 sequencing reads classified as bacterial were assigned to *Neisseria meningitidis*. In line with these data, mapping of reads against clade-specific and 16S rRNA gene markers revealed *N*. *meningitidis* as the most represented species. Assembled metagenomic fragments had the best average nucleotide identity (ANI) with *N*. *meningitidis*. Comparison of assembled contigs to reference alleles showed that this strain belongs to the ST-41/44 complex. *N. meningitidis* is commonly associated with meningitis and/or septicemia but should not be neglected as a causative agent of IE, which became exceedingly rare with the introduction of antibiotics. Our data show that WMGS may be used as a diagnostic procedure to strengthen the diagnosis of IE and to obtain draft genomic sequence of the pathogen and typing information.

## Background

Infective endocarditis (IE) remains a deadly disease despite significant progress in the diagnostic procedures and therapies. The most common cases of IE involve staphylococci, streptococci and enterococci as the preponderant microorganisms (>88%) recovered from blood cultures (1). The HACEK group (*Haemophilus* spp., *Aggregatibacter* spp., *Cardiobacterium* spp., *Eikenella corrodens*, and *Klebsiella* spp.) of fastidious oropharyngeal bacteria and other gram-negative organisms were reported to cause infection in 0.9–1.6% and 7.4% of IE patients with positive blood culture, respectively (1, 2).

A recent study reported that in 31% of the 918 cases suspected for IE (1), no causative microorganism could be grown using standard blood culture methods. Nevertheless, causative agents can be identified in ~50% of blood culture-negative patients (3) using other methods, including serology, the analysis of the excised valve by microscopy and PCR, and culture-based analyses of samples from the excised valve and other body sites. To increase the probability of pathogen identification, additional diagnostic tests have been proposed such as immunohistology, blood PCR, culture on specific media, prolonged culture period and tissue culture (4, 5). In 70–80% of the patients for whom pathogens remained unidentified by culture and other procedures, antibiotics had been given before the blood was taken for culture (3, 6).

Identifying the causative agent of IE is mandatory to initiate an appropriate antibiotic regimen. To increase the likelihood of identifying the pathogen, it is recommended to take three sets of blood cultures before an antibiotic therapy starts (7). The initial choice of empirical therapy is guided by many factors such as the nature (native or prosthetic) of the infected valve, the exposure to previous antibiotic therapy and the epidemiological type (community or nosocomial) of the infection.

Our case reports an unusual community-acquired blood-culture negative IE due to *Neisseria meningitidis* and details the next-generation sequencing (NGS) analysis performed to support this rare diagnosis.

## Case Report

A 54-year-old woman presented to the emergency department with dyspnea associated with cough and fever. Her past medical history included allergic asthma and an episode of pulmonary embolism 2 months prior to this hospitalization.

Dyspnea began 2 weeks before her arrival at the hospital. Because of the worsening of symptoms with the development of fever, the patient visited a general practitioner who diagnosed community-onset pneumonia and prescribed co-amoxicillin.

The next morning, she presented at the emergency department of our hospital because of increased dyspnea. Physical examination revealed sinus tachycardia at 110/min, central temperature of 39.2°C and a normal blood pressure of 113/69 mmHg. Cardiac examination showed a systolic murmur maximal at the aortic area and chest auscultation was compatible with left congestive heart failure. She had otherwise no systemic stigmata of infective endocarditis.

Blood tests revealed white blood cell 20.2 × 10^9^/l (neutrophils 15.86 × 10^9^/l, no left shift), hemoglobin 100 g/l, thrombocytes 407 × 10^9^/l, urea 6.4 mmol/l, creatinine 91 μmol/l, C-reactive protein 119 mg/l. The coagulation tests were abnormal but difficult to interpret, since she was taking rivaroxaban for the last 2 months. Two sets (aerobic and anaerobic) of blood cultures were carried out (BD BACTEC™ FX blood culture system) on days 0 and 1.

A chest X-ray was suggestive of a pneumonia at the right pulmonary base but also revealed signs of congestive heart failure. The patient was admitted to the floor and an antibiotic therapy with co-amoxicillin and clarithromycin was started for a severe community acquired pneumonia. An electrocardiogram showed a first-degree heart block without other abnormalities.

Shortly after admission, the patient presented an oppressive chest pain that resolved spontaneously. The electrocardiogram showed flattened T waves in all derivations with the exception of V5-V6 where they were inverted. Cardiac troponin (measured by a high sensitivity assay) was elevated at 112 ng/l. Total creatine kinase was normal with a concentration of 97 U/l, with no significant changes found by repeating the measurement after a 4-h interval.

The patient was admitted to the intensive care unit and intubated. The transesophageal echocardiography (TEE) and transthoracic echocardiography (TTE), performed at day 1, showed an echo density of 14 mm on the left coronary leaflet of the aortic valve and signs suggestive of a ruptured abscess of the mitro-aortic junction that caused a severe aortic regurgitation.

Antibiotic therapy for severe community acquired pneumonia was replaced by ceftriaxone and gentamicin to empirically manage infective endocarditis due to penicillin-sensitive or -resistant streptococci (5). At day 2, after performing a full body and coronary computed tomography scan that did not show secondary lesions nor coronary pathology, the patient underwent a semi-urgent aortic valve replacement by a mechanical prosthetic valve. The cardiac abscess cavity was closed by a pericardial patch.

The cardiac surgeon reported that the valve had an unusual exophytic structure, like a cauliflower. The histological examination of the valve showed a myxoid degeneration of the leaflets with the presence of vegetations and no bacterial or fungal elements evidenced by Gram and periodic acid–Schiff (PAS) staining.

The blood cultures remained sterile 5 days after admission. In the meantime (day 4), the patient received in addition to ceftriaxone and gentamycin, doxycycline for empirical coverage of *Coxiella burnetti, Brucella* spp., and *Bartonella henselae*. The serologies for those pathogens revealed negative at day 13. A culture of the valve remained sterile on day 8, after a 6-day incubation. A broad range PCR on the valve tissue showed (day 5) the presence of *Neisseria* sp., that was confirmed by specific qPCR assays leading to the identification of *N. meningitidis* serogroup B by targeting *ctrA* (8), *sodC* (9), *tauE, metA, shlA* (10), and *siaD* markers (11) on day 9. A search for hypocomplementemia showed normal levels of C3 and C4.

The antibiotic regimen was changed (day 13) for ceftriaxone alone for a total duration of 6 weeks.

## Discussion

In this case report, the initial diagnosis of pneumonia led to some delay in diagnosing community-acquired IE. Retrospectively, we can attribute the symptoms of acute dyspnoea and fever to the IE complicated by an acute aortic valve insufficiency.

The fact that blood cultures remained sterile may be ascribed to the administration of one dose of co-amoxicillin prior to the patient's hospitalization and possibly the fastidious nature of the microorganism. The atypical clinical presentation associated with a pneumonia, made us suspect an infection due to *Chlamydia pneumoniae, Mycoplasma pneumoniae*, or *Legionella pneumophila* in addition to common IE-causing gram-positive cocci and bacteria from the HACEK group.

We tested the suitability of whole metagenomic shotgun sequencing, as a method that does not target specific species, for use in diagnosing bacterial infections (Supplementary Material). We extracted DNA from a 30-mg heart valve specimen using a procedure that enables enrichment of microbial DNA and therefore better assessment of bacterial community by NGS. The concentration of bacterial DNA in the sample extract (4.4 pg/μL) was roughly 10-fold lower than human DNA concentration (45.69 pg/μL), and ~20-fold higher than bacterial DNA concentration in the negative control (NEC) (0.23 pg/μL), as assessed by qPCR experiments.

**Figure 1:**
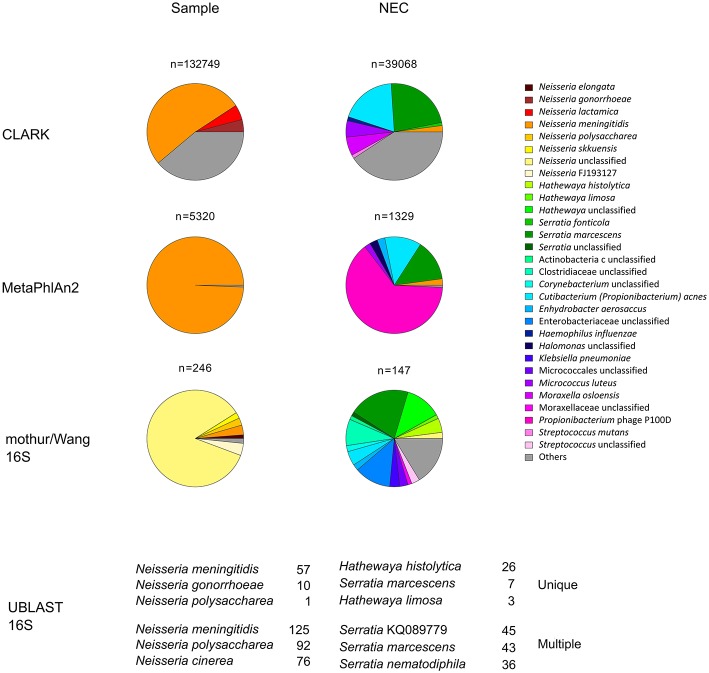
Relative abundance of prokaryotic taxa in metagenomic dataset. Proportions were determined by classifying at the species level sequences of genome fragments (CLARK), read mapping to clade markers (MetaPhlAn2) or by classifying 16S rRNA gene fragment sequences (mothur/Wang 16S and UBLAST 16S). All taxa with relative abundance <1% were summed up and represented as “others.” For simplicity, in the mothur/Wang 16S approach, only the results for the forward sequence reads are presented. They were further analyzed by UBLAST-alignments to the EzBioCloud reference 16S database (UBLAST 16S) and the top three most frequently identified species based on unique or multiple UBLAST hits are indicated. n, number of hits obtained for each sample and method used; NEC, negative extraction control.

Illumina sequencing followed by quality control checks, yielded 1,709,287 and 166,806 reads pairs for the sample and NEC metagenomic libraries, respectively. Most of the 103,136 reads classified by CLARK as bacterial in the sample data set were assigned to *Neisseria* (98.81%) at the genus level and, at the species level, to *N. meningitidis* (51.91%) (Figure 1). The most abundant organism from the genera other than *Neisseria* was *Cutibacterium* (*Propionibacterium*) *acnes*, a known reagent contaminant (12). Mapping of reads against clade-specific markers using MetaPhlAn2 revealed only two species: highly dominant *N*. *meningitidis* (99.38%) and unclassified Propionibacteriaceae (0.62%). In line with these analyses, the majority of reads mapping to 16S rRNA genes were classified to genus *Neisseria* with a k-mer based (Wang) approach and EzBioCloud reference 16S database. Taxonomic assignments of metagenomic 16S rRNA gene fragments, based on UBLAST-alignments, showed that both unique and multiple top hits were by far most frequently assigned to *N*. *meningitidis*.

Organisms other than *N*. *meningitidis* dominated the negative control. Small percentage of *Neisseria* reads identified in negative controls may be ascribed to cross-contamination during DNA extraction or sequencing library preparation, or to assignment of sequence reads to the wrong index during demultiplexing. By combining the results of bacterial DNA quantification from qPCR experiments with the relative abundance of bacterial taxa obtained by NGS (13), we estimated that the *Neisseria* DNA load in NEC was about 430 and 890 times lower than that of the valve specimen extract, for CLARK and MetaPhlAn2-based analyses, respectively.

**Figure 2:**
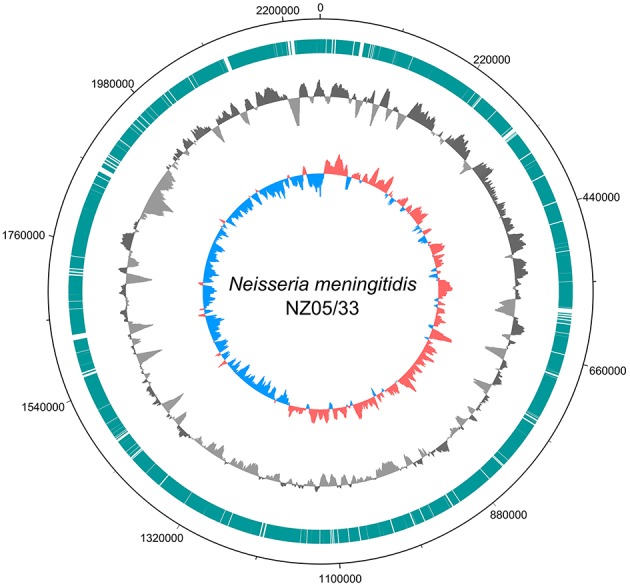
Ring diagram showing the genome of the reference *N*. *meningitidis* strain NZ-05/33 chromosome sequence (GenBank accession number NC_017518.1) and homologous metagenome sequences from this study. Circles depict (from outside to inside): chromosomal position; chromosomal regions to which mapped selected (classified by CLARK as belonging to genus *Neisseria*) metagenomic reads (turquoise bars); sequencing depth of the chromosomal regions obtained by mapping selected metagenomic reads (dark gray for above-average and light gray for below-average) using a 10,000 nt window in steps of 200 nt; GC skew with a 10,000 nt window in steps of 200 nt (red for positive, blue for negative). The figure was generated using DNAPlotter (14).

The reads from the sample dataset assigned by CLARK to genus *Neisseria* were assembled into 362 contigs. The evaluation of assembled contigs with QUAST revealed that they covered 88.1% of the reference NZ-05/33 genome. The metagenomic fragments that mapped to the reference genome sequence (Figure 2) were more represented in the origin-half of the chromosome. Similar observations was made for *Brucella melitensis* DNA fragments found in a necrotic hepatic lesion of a patient with a chronic brucelloma (15). Such a pattern suggests that the infective agent was actively replicating (16, 17).

Assembled sequence contigs had average nucleotide identity (ANI) values of 97.03–99.47% with 1,368 *N*. *meningitidis*, 94.76–95.02% with 473 *N*. *gonorrhoeae*, and ≤94.52% with other *Neisseria* species isolates whose genomic sequences were available in the NCBI database. Comparison of assembled contigs to reference alleles from the *Neisseria* PubMLST database showed that the *Neisseria* strain in question perfectly matched (100% identity and 100% alignment) the seven MLST alleles (*abcZ*_3, *adk*_6, *aroE*_34, *fumC*_5, *gdh*_22, *pdhC*_6, *pgm*_9) of the ST-41/44 complex. This clonal complex is by far most frequently associated with serogroup B strains (18, 19).

Mapping of contigs to the ResFinder database did not reveal any acquired antimicrobial resistance gene but identified an aspartate at position 121 of the porin *porB* gene, that in *N*. *gonorrhoeae* contributes to a partial resistance to penicillins, tetracyclines, and cephalosporins (20).

## Conclusion

The most common pathogen among *Neisseria* species causing IE remain *Neisseria gonorrhoeae*, with 71 cases reported since 1939, involving predominantly left-sided native valves in young males (21, 22), and *Neisseria elongata* with 18–24 cases (23, 24) described in literature. *N*. *meningitidis* can cause a broad spectrum of clinical manifestations and is largely known for worldwide diseases, such as purulent meningitis and/or septicemia associated with high morbidity and mortality. IE caused by *N*. *meningitidis* was more common before the antibiotic era with a trend of affecting the left side of the heart. Since 1960s, meningococcal IE has become a rare disease with only 13 reported cases (25). Nevertheless, *N*. *meningitidis* should not be neglected as a causative agent of IE. Our data show that whole metagenome shotgun sequencing (WMGS) may be used as a diagnostic procedure to strengthen the diagnosis of IE, especially in culture-negative cases, and to obtain draft genomic sequence of the pathogen and typing information.

## Data Availability

The datasets generated for this study can be found in European Nucleotide Archive (ENA), PRJEB24753.

## Ethics Statement

According to hospital protocol, no formal ethics approval was required. The patient agreed and provided written informed consent for publication of this case report.

## Author Contributions

VC, VL, CH, and JS analyzed and interpreted patient data. MG, NG, SL, GR, and PK performed the experiments. NG, SL, and VL analyzed the metagenomics data. VC, VL, and JS wrote the manuscript. All authors read and approved the final manuscript.

### Conflict of Interest Statement

The authors declare that the research was conducted in the absence of any commercial or financial relationships that could be construed as a potential conflict of interest.
