# Supplementary material for: Rare Case of Community-Acquired Endocarditis Caused by Neisseria meningitidis Assessed by Clinical Metagenomics
Source: Front Cardiovasc Med. 2019 Aug 6;6:112. doi: 10.3389/fcvm.2019.00112 (PMC6691042; doi:10.3389/fcvm.2019.00112)
Supplement: Supplementary file 1 [file Data_Sheet_1.pdf]

Clinical metagenomics and genomics methods

DNA extraction. Thirty mg of fresh cardiac valve tissue was cut with a scalpel into small pieces on a disposable Petri dish, mixed with 10 µL of DPBS buffer (Sigma) and homogenized with an Omni Hard Tissue Homogenizing Mix (2-mL tubes) on a Bead Ruptor 4 (Omni) at speed 3 for 30 seconds. The volume of the homogenate was adjusted to 200 µL with DPBS. DNA was extracted with the Ultra-Deep Microbiome Prep kit (Molzym) according to the manufacturer's instructions for tissue samples. Purified DNA was eluted in 100 µL and stored at –20°C. Negative extraction control was performed by using 200 µL of the DPBS Buffer (Sigma) instead of the clinical sample.

qPCR assays. Human and bacterial DNA concentrations were determined by qPCR experiments targeting beta-actin and 16S rRNA reference genes, respectively, as described previously (Lazarevic et al., 2014). For easier comparison of bacterial and human DNA load, we converted the number of copies of the 16S rRNA gene to DNA mass by considering that 1,493 copies of the 16S rRNA gene correspond to 1 pg DNA (a figure valid for *Escherichia coli* strain DH5-alpha whose genomic DNA was used to construct a reference curve).

DNA sequencing. The Nextera XT (Illumina, San Diego, USA) metagenomic libraries were prepared from 5 µL of DNA eluates, containing 250 pg (valve sample) or 1.15 pg (negative control) DNA (sum of bacterial and human DNA loads determined by qPCR) using 18 PCR enrichment cycles. The libraries were sequenced (2×250) at Fasteris (Plan-les-Ouates) on an Illumina MiSeq instrument with the MiSeq Reagent Kit v3 (Illumina).

Bioinformatics analysis. Trimmomatic v.0.36 package (Bolger et al., 2014) was used to: (i) remove Illumina adapter sequences, (ii) cut off leading and trailing sequences at bases under the Phred quality threshold of 5 and (iii) to trim low-quality ends of reads at the beginning of any 20-base wide sliding window with an average Phred quality <30. The reads that were <150 bases in length after trimming were discarded. Putative artifactual replicate reads were filtered out using a homemade script that retains the longest sequence among those with identical first hundred bases, in either forward or reverse reads. Any forward or reverse reads without its corresponding paired read were discarded. To control for human DNA contamination, we removed all reads assigned to the phylum Chordata (to which belongs the species *Homo sapiens*) based on the CLARK (Ounit et al., 2015) v.1.2.3.2 classification at the phylum level with the default parameters against human genome sequence (GRCh38.p7) (International Human Genome Sequencing Consortium, 2001; Pruitt et al., 2007). Remaining data were deposited to European Nucleotide Archive (ENA) database (accession number PRJEB24753) and analyzed. The reads were classified at the species, genus and phylum levels using CLARK (with parameters -m 0 -c 0.8) against a collection of 251 archaeal, 5,166 bacterial and 4,414 viral genomes from the NCBI/RefSeq database (20 November 2017) (Pruitt et al., 2007). The prokaryotic genomes used to build the CLARK database were selected and curated by removal of ambiguous contigs as described by Kirstahler et al. (2017). The percentage of each prokaryotic species or genus was determined relative to the total number of prokaryotic reads identified at the phylum level.

Reads corresponding to bacterial and archaeal 16S rRNA genes were selected using USEARCH (Edgar, 2010) 8.1.1861 (-usearch_local -id 0.9 -query_cov 1 -top_hit_only -strand both) against EzBioCloud 16S database (Yoon et al., 2017) (downloaded on September 5, 2017). The selected reads were classified using two methods: (i) mothur's v.1.39.5 (Schloss et al., 2009) command classify.seq with Wang approach (Wang et al., 2007) at 0.8 confidence threshold and the EzBioCloud 16S database, and (ii) by mapping the reads against the EzBioCloud 16S database with the USEARCH software (-ublast -id 0.95 -evalue 0.00001 -strand both

-top_hits_only). With this approach, we discriminated the reads with single from those with multiple best hits.

MetaPhlAn2 (Truong et al., 2015) taxonomic profiling, based on mapping of reads against clade markers, was used with default settings.

Assembly and analysis of the N. meningitidis draft genome recovered from the metagenomic data. After removal of low quality reads and reads matching human genome (as described above), the reads from a non-dereplicated dataset were classified at the genus level with CLARK. The reads assigned to the genus *Neisseria* were selected and mapped to the reference *N. meningitidis* NZ-05/33 chromosome using BWA (Li and Durbin, 2009) v.0.7.17 with default parameters. These selected reads were assembled with SPAdes (Bankevich et al., 2012) (-k 21,33,55,77,99,127 --careful) followed by QUAST v.4.3 (Gurevich et al., 2013) evaluation. Average Nucleotide Identity (ANI) between the draft genome assembly and NCBI *N. meningitidis* genome sequences (assessed on June 5, 2018) was calculated using pyani (Pritchard et al., 2016) with BLAST (Altschul et al., 1990) method. Multilocus sequence typing (MLST) analysis was performed by mapping contigs against the *Neisseria* MLST database (Jolley and Maiden, 2010) from the http://pubmlst.org/neisseria site (downloaded on February 6, 2018) with SeqSphere+ (Ridom) software v.3.1.0 with minimum sequence identity of 90% and minimum alignment or 99%. Acquired antimicrobial resistance genes (using an identity threshold of 90% and target coverage of 100%) and chromosomal mutations in the draft genome assembly were searched for with ResFinder 3.0 (Zankari et al., 2012) (http://cge.cbs.dtu.dk/services/ResFinder).

References

Altschul, S.F., Gish, W., Miller, W., Myers, E.W., and Lipman, D.J. (1990). Basic local alignment search tool. *Journal of Molecular Biology* 215(3), 403-410. doi: 10.1006/jmbi.1990.9999 S0022283680799990 [pii].

Bankevich, A., Nurk, S., Antipov, D., Gurevich, A.A., Dvorkin, M., Kulikov, A.S., et al. (2012). SPAdes: A new genome assembly algorithm and its applications to single-cell sequencing. *Journal of Computational Biology* 19(5), 455-477. doi: 10.1089/cmb.2012.0021.

Bolger, A.M., Lohse, M., and Usadel, B. (2014). Trimmomatic: a flexible trimmer for Illumina sequence data. *Bioinformatics* 30(15), 2114-2120. doi: 10.1093/bioinformatics/btu170.

Edgar, R.C. (2010). Search and clustering orders of magnitude faster than BLAST. *Bioinformatics* 26(19), 2460-2461. doi: 10.1093/bioinformatics/btq461.

Gurevich, A., Saveliev, V., Vyahhi, N., and Tesler, G. (2013). QUAST: quality assessment tool for genome assemblies. *Bioinformatics* 29(8), 1072-1075. doi: 10.1093/bioinformatics/btt086.

International Human Genome Sequencing Consortium (2001). Initial sequencing and analysis of the human genome. *Nature* 409, 860. doi: 10.1038/35057062.

Jolley, K.A., and Maiden, M.C.J. (2010). BIGSdb: Scalable analysis of bacterial genome variation at the population level. *BMC Bioinformatics* 11, 595-595. doi: 10.1186/1471-2105-11-595.

Kirstahler, P., Solborg Bjerrum, S., Friis-Moller, A., la Cour, M., Aarestrup, F.M., Westh, H., et al. (2018). Genomics-based identification of microorganisms in human ocular body fluid. *Scientific Reports* 8(1), 4126. doi: 10.1038/s41598-018-22416-4.

Lazarevic, V., Gaïa, N., Emonet, S., Girard, M., Renzi, G., Despres, L., et al. (2014). Challenges in the culture-independent analysis of oral and respiratory samples from intubated patients. *Frontiers in Cellular and Infection Microbiology* 4, 65. doi: 10.3389/fcimb.2014.00065.

Li, H., and Durbin, R. (2009). Fast and accurate short read alignment with Burrows–Wheeler transform. *Bioinformatics* 25(14), 1754-1760. doi: 10.1093/bioinformatics/btp324.

Ounit, R., Wanamaker, S., Close, T.J., and Lonardi, S. (2015). CLARK: fast and accurate classification of metagenomic and genomic sequences using discriminative k-mers. *BMC Genomics* 16, 236. doi: 10.1186/s12864-015-1419-2.

Pritchard, L., Glover, R.H., Humphris, S., Elphinstone, J.G., and Toth, I.K. (2016). Genomics and taxonomy in diagnostics for food security: soft-rotting enterobacterial plant pathogens. *Analytical Methods* 8(1), 12-24. doi: 10.1039/c5ay02550h.

Pruitt, K.D., Tatusova, T., and Maglott, D.R. (2007). NCBI reference sequences (RefSeq): a curated non-redundant sequence database of genomes, transcripts and proteins. *Nucleic Acids Research* 35(Database issue), D61-D65. doi: 10.1093/nar/gkl842.

Schloss, P.D., Westcott, S.L., Ryabin, T., Hall, J.R., Hartmann, M., Hollister, E.B., et al. (2009). Introducing mothur: open-source, platform-independent, community-supported software for describing and comparing microbial communities. *Applied and Environmental Microbiology* 75(23), 7537-7541. doi: 10.1128/AEM.01541-09.

Truong, D.T., Franzosa, E.A., Tickle, T.L., Scholz, M., Weingart, G., Pasolli, E., et al. (2015). MetaPhlAn2 for enhanced metagenomic taxonomic profiling. *Nature Methods* 12(10), 902-903. doi: 10.1038/nmeth.3589.

Wang, Q., Garrity, G.M., Tiedje, J.M., and Cole, J.R. (2007). Naive Bayesian classifier for rapid assignment of rRNA sequences into the new bacterial taxonomy. *Applied and Environmental Microbiology* 73(16), 5261-5267. doi: 10.1128/AEM.00062-07.

Yoon, S.-H., Ha, S.-M., Kwon, S., Lim, J., Kim, Y., Seo, H., et al. (2017). Introducing EzBioCloud: a taxonomically united database of 16S rRNA gene sequences and whole-genome assemblies. *International Journal of Systematic and Evolutionary Microbiology* 67(5), 1613-1617. doi: 10.1099/ijsem.0.001755.

Zankari, E., Hasman, H., Cosentino, S., Vestergaard, M., Rasmussen, S., Lund, O., et al. (2012). Identification of acquired antimicrobial resistance genes. *Journal of Antimicrobial Chemotherapy* 67(11), 2640-2644. doi: 10.1093/jac/dks261.
